# Thinking false and slow: Implausible beliefs and the Cognitive Reflection Test

**DOI:** 10.3758/s13423-023-02321-2

**Published:** 2023-06-27

**Authors:** Kristy A. Martire, Samuel G. Robson, Manisara Drew, Kate Nicholls, Kate Faasse

**Affiliations:** The University of New South Wales, Kensington, NSW 2052 Australia

**Keywords:** Fake news, Analytic thinking, Cognitive reflection test, Intuition, Misinformation, Conspiracy theories

## Abstract

Why do people believe implausible claims like conspiracy theories, pseudoscience, and fake news? Past studies using the Cognitive Reflection Test (CRT) suggest that implausible beliefs may result from an unwillingness to effortfully process information (i.e., cognitive miserliness). Our analysis (N = 664) tests this account by comparing CRT performance (total score, number and proportion of incorrect intuitive responses, and completion time) for endorsers and non-endorsers of implausible claims. Our results show that endorsers performed worse than non-endorsers on the CRT, but they took significantly *longer* to answer the questions and did not make proportionally more intuitive mistakes. Endorsers therefore appear to process information effortfully but nonetheless score lower on the CRT. Poorer overall CRT performance may not necessarily indicate that those who endorse implausible beliefs have a more reflexive, intuitive, or non-analytical cognitive style than non-endorsers.

**Supplementary Information:**

The online version contains supplementary material available at 10.3758/s13423-023-02321-2.

## Introduction

Information is more accessible now than ever before. Most of us carry an inexhaustible supply of truths, errors, exaggerations, and outright lies with us in our pockets every day. However, the human capacity to process and evaluate the credibility of this information is limited and can lead to errors in thinking and judgment (Hills, 2019). As a result, some people come to believe things that are highly implausible given current scientific knowledge or logic, including conspiracy theories, fake news, and pseudoscience. These beliefs can have potentially dire consequences when they relate to critical issues (Dyer & Hall, 2019; Lewandowsky, 2021; Lobato et al., 2014; van der Linden, 2015). For example, belief in vaccine conspiracies is related to negative vaccination attitudes and negatively predicts COVID-19 and other vaccine intentions (Bertin et al., 2020). It is therefore vitally important to understand who among us is most susceptible to implausible beliefs and how that vulnerability can potentially be reduced.

Understanding what makes people susceptible to implausible claims is a topic that has received a great deal of attention. However, research in this area is largely correlational because it is impractical to randomly allocate participants to hold any particular belief. Studies typically examine whether there is an association between implausible beliefs and various psychological constructs, (e.g., Bertin et al., 2020; Lobato et al., 2014; Pennycook et al., 2012; Pennycook & Rand, 2019, 2020; Ross et al., 2021; Scherer et al., 2021; Ståhl & van Prooijen, 2018; van Prooijen, 2017). Findings suggest that people who endorse one implausible belief are more likely to hold other implausible beliefs, to believe in simple solutions to complex problems, and to be more dogmatic, delusion-prone, and more convinced by pseudo-profound statements (Bronstein et al., 2019; Lobato et al., 2014; Pennycook & Rand, 2020; van Prooijen, 2017). They also show less open-minded thinking, less knowledge about science, and less belief in climate science or the idea that truth is knowable (Lewandowsky, 2021; Lewandowsky et al., 2013; Pennycook et al., 2020; Swami et al., 2014).

Relative to non-endorsers, those who endorse implausible claims also tend to perform worse on the Cognitive Reflection Test (CRT; Bago et al., 2020; Bronstein et al., 2019; Ballová Mikušková & Čavojová, 2020; Patel et al., 2019; Pennycook et al., 2012; Pennycook et al., 2020; Pennycook & Rand, 2019, 2020; Rizeq et al., 2021; Ross et al., 2021; Scherer et al., 2021; Shenhav et al., 2012; Ståhl & van Prooijen, 2018; van Prooijen, 2017). The CRT was designed as a three-item test (Frederick, 2005; Table 1, Items 1–3) to capture a tendency to override a tempting but inaccurate ‘intuitive’ answer to a problem through reflective, analytic effort (see Table 1, Items 4–7 for other item variants by Thomson & Oppenheimer, 2016). Scoring typically involves summing the number of correct answers. Lower CRT scores have been viewed as synonymous with lazy thinking (Pennycook et al., 2016; Toplak et al., 2011), and thus people who more strongly endorse implausible claims have been characterized as intuitive, uncritical, unreflective, non-analytic thinkers (Bago et al., 2020; Ballová Mikušková & Čavojová, 2020; Pennycook et al., 2012; Pennycook & Rand, 2019; Shenhav et al., 2012). In other words, people who endorse implausible claims are cognitive misers (Toplak et al., 2011).

**Table 1:** Cognitive Reflection Test questions and responses

Item	Question	Correct answer	Intuitive answer
1	A bat and a ball cost $1.10 in total. The bat costs $1.00 more than the ball. How much does the ball cost?	5 cents	10 cents
2	If it takes 5 machines 5 minutes to make 5 widgets, how long would it take 100 machines to make 100 widgets?	5 minutes	100 minutes
3	In a lake, there is a patch of lily pads. Every day, the patch doubles in size. If it takes 48 days for the patch to cover the entire lake, how long would it take for the patch to cover half of the lake?	47 days	24 days
4	How many cubic feet of dirt are there in a hole that is 3' deep x 3' wide x 3' long?	None	27 cubic feet of dirt
5	A farmer had 15 sheep and all but 8 died. How many are left?	8	7
6	Emily’s father has three daughters. The first two are named April and May. What is the third daughter's name?	Emily	June
7	If you’re running a race and you pass the person in second place, what place are you in?	Second	First

However, the assumption that the CRT measures miserly thinking has been questioned (Stupple et al., 2017). Some have argued that CRT performance does not necessarily reflect a thinking style (Blacksmith et al., 2019) nor a capacity to override intuitive responses (Erceg et al., 2020). A recent quasi-experimental analysis also raises questions about the miserly explanation for implausible beliefs; secondary analysis of evidence evaluations revealed that endorsers were more easily persuaded by evidence than non-endorsers, but were *not less sensitive* to evidence quality (Martire et al., 2020). This result is only possible if endorsers engage in an effortful analysis of the information presented.

### The present study

In this quasi-experimental study, we test the ‘miserly’ explanation for implausible beliefs by examining how CRT performance differs between ‘endorsers’ and ‘non-endorsers’ of implausible claims. Does a lack of effort account well for the differences between these groups? One can easily compute the number of correct responses on the CRT (CRT-Reflective score) and the number of incorrect intuitive (or lure) responses (e.g., saying 10 cents on the bat-and-ball problem; CRT-Intuitive; Pennycook et al., 2016). However, cognitive effort on this task may be confounded by cognitive ability (see Blacksmith et al., 2019; Sinayev & Peters, 2015; Sirota et al., 2021; Stupple et al., 2017; Thomson & Oppenheimer, 2016), and the Intuitive and Reflective scores are often strongly inversely related so as to be virtually indistinguishable (Blacksmith et al., 2019; Erceg & Bubić, 2017; Pennycook et al., 2016). To determine if endorsers are truly lazy thinkers, one can shift attention to the incorrect responses. A more intuitive person ought to provide a greater proportion of intuitive incorrect responses relative to ‘other’ incorrect responses (e.g., saying 10 cents vs. 15 cents on the bat-and-ball problem; CRT-Proportion Intuitive [PI]; Pennycook et al., 2016). Because this score is derived from within-group errors, it is independent of the Reflective score, and is therefore less likely to be confounded by cognitive ability. Total and item response times can also capture a person’s effort when answering CRT problems. A correct response does not always require lengthy deliberation (see Bago & De Neys, 2019), but it is difficult to imagine how more analytic, effortful thinking would elicit quicker responses than a reflexive, intuitive approach (Stupple et al., 2017).

As a result, we expect endorsers of implausible claims to perform more poorly than non-endorsers on the CRT (lower CRT-Reflective score), and to have higher CRT-Intuitive scores given that these two measures are often highly correlated. However, if the miserly account is correct, we expect endorsers to make a higher proportion of intuitive mistakes (CRT-PI score) and complete the CRT faster than non-endorsers. Conversely, if endorsers are engaging effortfully in the task, we would not expect them to make proportionally more incorrect intuitive mistakes than non-endorsers, nor complete the CRT faster than non-endorsers.

## Methods

### Design and materials

This experiment was approved by the University of New South Wales Human Research Ethics Approval Panel – C, File 3452, and was preregistered (AsPredicted #61190 https://aspredicted.org/55qg8.pdf). Materials, data, and code for this study can be accessed online via the Open Science Framework at: https://osf.io/uyw98/.

We employed a one-way quasi-experimental design where implausible belief endorsement (endorser or non-endorser) varied between subjects. Implausible beliefs vary across the population and are typically measured in terms of degree (e.g., Swami et al., 2010; Tobacyk & Milford, 1983). However, normative samples may include only very few people who strongly endorse a highly implausible claim and thus may not adequately represent those with the most potentially problematic beliefs. To address this, we asked participants to rate their level of belief on a scale from 0 (not at all) to 100 (definitely) on the following three highly implausible items: (1) *Vaccines are harmful and this fact is covered up* (Jolley & Douglas, 2014), (2) *Global warming is a hoax* (van der Linden, 2015), or (3) *The earth is flat*. We reasoned that few people would give credence to these large-scale conspiratorial or highly implausible claims and that our data would be non-normally distributed, so we dichotomized participants into groups. We defined endorsers in our primary analyses as those who rated one or more of the three implausible claims: ≥ 75 on a scale from 0 ‘not at all’ to 100 ‘definitely true’. Non-endorsers were those who rated all three claims < 50. Logically speaking, endorsers of such highly implausible claims should be the most intuitive if intuitive thinking is a key mechanism that underlies implausible beliefs. Participants who rated all three items between 50 and 75 were excluded from analysis because these potentially ambivalent responses are not strongly representative of those at either end of the implausible beliefs spectrum.

One additional implausible claim: *The Apollo moon landings never happened and were staged in a Hollywood film studio* (Lewandowsky et al., 2013), was added after preregistration and in the wake of widespread public discourse on vaccines during the COVID pandemic. This item was only used to define endorsers in one of our post hoc analyses.

Because we reasoned that many people would be unlikely to endorse these highly implausible claims, we decided to oversample people who we knew believed in an implausible claim to provide the greatest chance of detecting genuine differences in terms of thinking style. Specifically, half of the participants we recruited had indicated that they believed in climate change when they signed up to Prolific (i.e., presumptive non-endorsers) and half indicated that they did not believe in climate change (i.e., presumptive endorsers). This item was a pre-screening tool and was not used to define endorsement for the preregistered primary analysis.

The dependent measures were: (1) CRT-Reflective score, (2) CRT-Intuitive score, (3) CRT-PI score, and (4) total completion time for a seven-item version of the CRT (Table 1; Frederick, 2005; Thomson & Oppenheimer, 2016). Each DV was calculated per participant as follows: CRT-*Reflective* score was the total number of correct responses out of a maximum of 7; CRT-*Intuitive* score was the total number of incorrect intuitive responses out of a maximum of 7; CRT-*PI* was the proportion of intuitive incorrect responses out of all incorrect responses. Total completion time was the sum of response times for the seven CRT items calculated from time of initial presentation to the submission of each response (Stupple et al., 2017).

### Participants

Based on Martire et al. (2020) and Stupple et al. (2017), we determined that 800 participants would be needed to generate a sufficient sample of endorsers and non-endorsers for analysis. Of the 800 participants we pre-screened, 400 stated that they *did* believe in climate change and 400 stated that they *did not* believe in climate change. After excluding participants based on predefined selection criteria and grouping people using the three implausible belief items above, 170 endorsers and 517 non-endorsers remained. However, we removed an additional 23 participants who had indicated that they used google/internet to answer the CRT questions, leaving 162 endorsers and 502 non-endorsers for the primary analysis.

On average participants were 39.43 years of age (range 18–79, *SD* = 13.81 years), most were male (53.9%), had a College/University degree (48.9%), identified as White/Caucasian (85.4%) and spoke English as their first language (71.8%). Of the 162 endorsers, 124 (74.3%) endorsed the claim that global warming was a hoax, 67 (41.3%) endorsed the claim that vaccines are harmful and the fact is covered up, and 21 (13.0%) endorsed a flat earth. Forty-two (25.1%) endorsed at least two claims and eight (4.9%) endorsed all three claims.

### Procedure

After providing informed consent, all participants were presented the CRT items online in a random order before answering questions about their familiarity with each item and completing a five-option multiple choice question asking participants to choose all options that described how they reached their answers to the CRT items: (1) *The answer to the question ‘jumped out’ at me*, (2) *I puzzled over the question before I worked out the answer*, (3) *I used Google/internet to find the answer*, (4) *I remembered the answer from having seen the question before*, (5) *None of these options describe how I reached my answers*. Options (1) and (2) served as a self-report measure of their decision-making approach, while options (3) and (4) served as data quality checks. Participants then completed a general knowledge test containing eight general knowledge items together with the four implausible claims described above (per Martire et al., 2020). Endorsement status was determined by responses to the implausible claims on the general knowledge test.

## Results

### Analysis of descriptives

Endorsers were significantly more likely to have completed only secondary education (32.7%) and were significantly less likely to have completed post-secondary education (i.e., College/University, Doctoral/Masters degree, Professional degree, or Trade qualification; 66.7%) than non-endorsers (secondary only: 24.1%; post-secondary: 75.5%), *χ*^2^_(1)_ = 4.77, *p* = .029. There was no significant association between gender or English fluency and endorsement status. However, endorsers were significantly older (*M* = 42.0, *SD* = 14.55) than non-endorsers (*M* = 38.6, *SD* = 13.48) *t*_(256.2)_ = 2.674, *p* = .008. See Table 2 for descriptive results.

**Table 2:** Results for planned and post hoc analyses comparing endorsers and non-endorsers of implausible claims

	Planned analysis	Post hoc analysis 1	Post hoc analysis 2	Post hoc analysis 3
Endorser definition:	Any of 3 claims ≥ 75	Any of 3 claims ≥ 75	Any of 4 claims ≥ 75	Disbelieves climate change
Non-endorser definition:	All of 3 claims < 50	All of 3 claims < 50	All of 4 claims ≤ 20	Believes in climate change
Additional exclusions:	Internet	Internet or remembered	Internet or remembered	Internet or remembered
***Descriptives***	Endorser; non-endorser	Endorser; non-endorser	Endorser; non-endorser	Endorser; non-endorser
N	162; 502	118; 298	129; 230	272; 223
Gender (% Male)	60.5; 51.8	54.2; 47.3	55.0; 48.7	55.9; 40.8
*Χ*^2^ statistic [*p*]	*χ*^2^_(1)_ = 3.678 [*p* =.055]	*χ*^2^_(1)_ = 1.522 [*p* =.217]	*χ*^2^_(1)_ = 1.144 [*p* =.285]	*χ*^2^_(1)_ = 10.813 [*p* =.001]
Highest education (% secondary)	32.7; 24.1	31.4; 21.5	32.6; 20.4	32.7; 17.9
*Χ*^2^ statistic [*p*]	*χ*^2^_(1)_ = 4.774 [*p* =.029]	*χ*^2^_(1)_ = 4.334 [*p* =.037]	*χ*^2^_(1)_ = 6.280 [*p* =.012]	*χ*^2^_(1)_ = 14.272 [*p* <.001]
English first language (% Yes)	69.8; 72.5	66.1; 69.1	65.1; 69.1	64.7; 71.7
*Χ*^2^ statistic [*p*]	*χ*^2^_(1)_ = 0.460 [*p* =.498]	*χ*^2^_(1)_ = 0.357 [*p* =.550]	*χ*^2^_(1)_ = 0.609 [*p* =.435]	*χ*^2^_(1)_ = 2.788 [*p* =.095]
Age (mean [SD])	42.0 [14.5]; 38.6 [13.5]	42.1 [15.0]; 38.4 [13.0]	41.1 [15.1]; 38.7 [13.0]	38.9 [14.9]; 39.5 [12.3]
*t* statistic [*p*]	*t*_(256.2)_ = 2.674 [*p* =.008]	*t*_(190.1)_ = 2.325 [*p* =.021]	*t*_(234.0)_ = 1.500 [*p* =.135]	*t*_(493.0)_ = -0.457 [*p* =.648]
***Hypothesis testing***				
CRT-reflective (median)	3; 5	3; 4	3; 4	3; 4
*W* statistic [*p*] *r*	*W* = 28310.00 [*p* <.001] *r* =.229	*W* = 12759.00 [*p* <.001] *r* =.216	*W* = 9984.00 [*p* <.001] *r* =.274	*W* = 25433.00 [*p* =.002] *r* =.140
CRT-intuitive (median)	3; 2	3; 2	3; 2	3; 2
*W* statistic [*p*] *r*	*W* = 51457.00 [*p* <.001] *r* =.200	*W* = 21755.00 [*p* <.001] *r* =.187	*W* = 18915.00 [*p* <.001] *r* =.231	*W* = 34737.00 [*p* =.005] *r* =.127
CRT-PI (median)	0.80; 0.83	0.82; 0.83	0.80; 0.83	0.83; 0.83
*W* statistic [*p*] *r*	*W* = 40256.00 [*p* =.839] *r* =.008	*W* = 17619.00 [p =.972] *r* =.002	*W* = 15028.00 [*p* =.829] *r* =.011	*W* = 31594.00 [*p* =.400] *r* =.038
CRT-total time (median)	182.59; 143.85	185.96; 158.02	184.36; 158.02	179.58; 150.07
*W* statistic [*p*] *r*	*W* = 48706.00 [*p* <.001] *r* =.147	*W* = 21160.00 [*p* =.001] *r* =.159	*W* = 17843.00 [*p* =.001] *r* =.168	*W* = 35486.00 [*p* =.001] *r* =.146
***Secondary analysis***				
‘Puzzled’ (% Yes)	72.8; 72.9	78.0; 78.2	78.3; 79.1	76.5; 79.8
*Χ*^2^ statistic [*p*]	*χ*^2^_(1)_ = 0.000 [*p* =.986]	*χ*^2^_(1)_ = 0.002 [*p* =.961]	*χ*^2^_(1)_ = 0.035 [*p* =.852]	*χ*^2^_(1)_ = 0.801[*p* =.371]
‘Jumped’ (% Yes)	32.1; 42.2	31.4; 39.6	31.0; 40.9	35.7; 40.8
*Χ*^2^ statistic [*p*]	*χ*^2^_(1)_ = 5.250 [*p* =.022]	*χ*^2^_(1)_ = 2.456 [*p* =.117]	*χ*^2^_(1)_ = 3.436 [*p* =.064]	*χ*^2^_(1)_ = 1.377[*p* =.241]

### Hypothesis testing

**Fig. 1:**
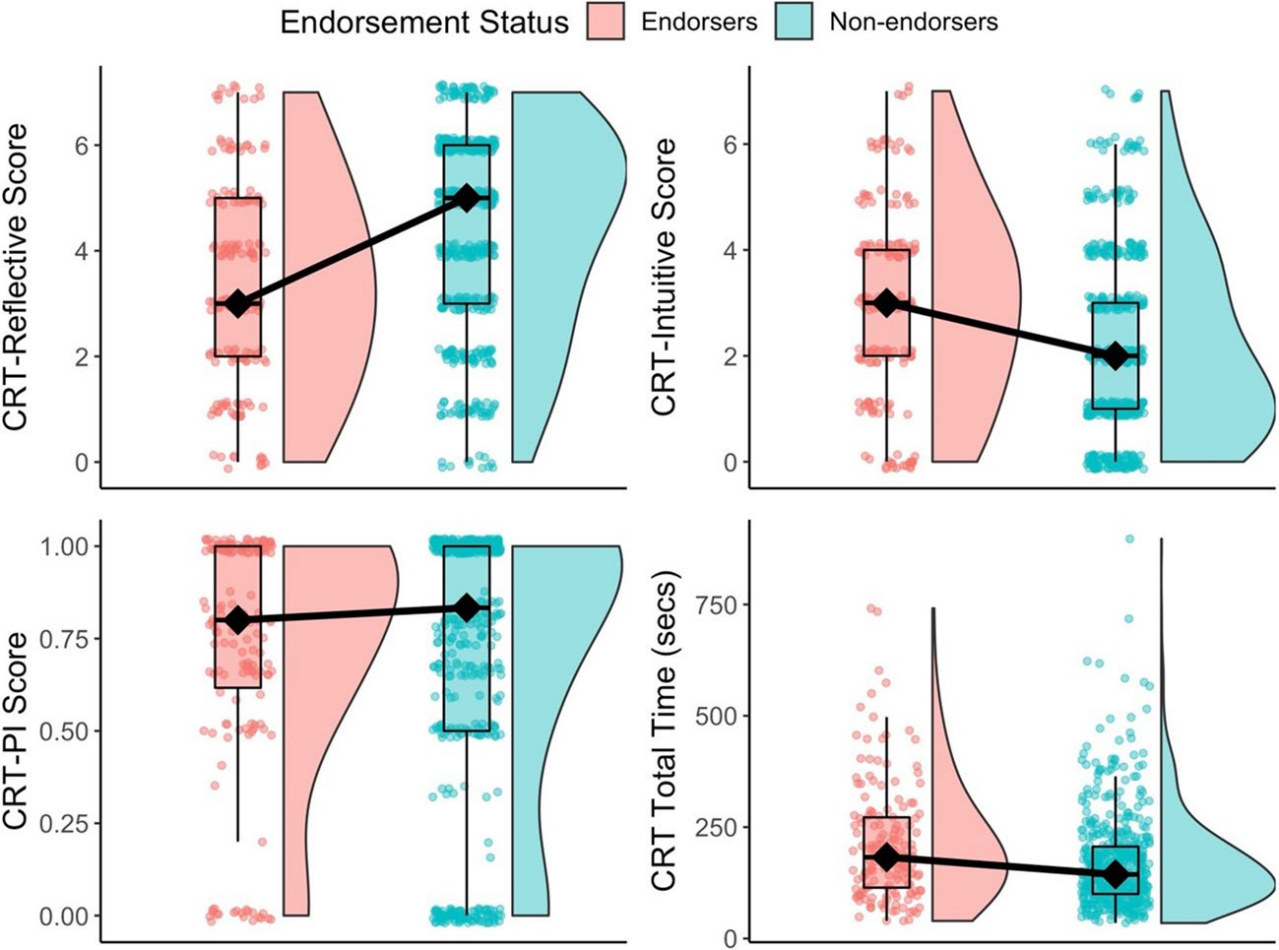
Raincloud plots showing the raw jittered data, box and whisker plots, and distribution of responses of endorsers and non-endorsers of implausible beliefs, for Cognitive Reflection Test (CRT)-Reflective, CRT-Intuitive, CRT-PI, and CRT-Total Time (left to right, top to bottom). The black lines connect the median response for each group (diamond). The box depicts the median, 25th and 75th percentiles. The whiskers extend from the hinge to the value no further than 1.5 x interquartile range above and below the upper and lower hinges. Endorsers were less accurate overall, were more likely to give incorrect intuitive answers, but took significantly longer to complete the CRT than non-endorsers. The proportion of incorrect intuitive answers out of all incorrect answers did not differ significantly between groups

Non-parametric two-tailed Mann-Whitney-Wilcoxon tests were conducted to compare endorsers and non-endorsers on CRT-Reflective scores, CRT-Intuitive scores, CRT-PI scores, and CRT Total completion time. This approach differed from the preregistered one-way multivariate analysis of variance (MANOVA) because the distribution of each outcome variable was significantly different from the normal distribution, making MANOVA inappropriate. In addition, the CRT-PI score calculation typically involves dividing the CRT-Intuitive score by the sum of all incorrect responses. This calculation results in missing values for participants with perfect performance and excludes them from the analysis (Erceg & Bubić, 2017; Pennycook et al., 2016). To avoid excluding participants, we decided to include those with perfect performance (given a value of 0), though this made little difference to the result. A summary of all results is presented in Fig. 1 and Table 2. The results of Bayesian t-tests, and several linear models with the average implausible belief score as the dependent variable, can be found in the Online Supplementary Materials (OSM).

#### CRT-Reflective scores

CRT-Reflective scores of endorsers (*M* = 3.35 *Mdn* = 3, *SD* = 1.96) were significantly lower than the CRT-Reflective scores of non-endorsers (*M* = 4.39, *Mdn* = 5, *SD* = 1.90), *W* = 28310.00, *p* < .001. The size of the effect was small* r* = .229. See Fig. 1, top-left panel.

#### CRT-Intuitive scores

Conversely, the CRT-Intuitive scores of endorsers (*M* = 2.94, *Mdn* = 3, *SD* = 1.87) were significantly higher than the scores of non-endorsers (*M* = 2.10, *Mdn* = 2, *SD* = 1.75), *W* = 51457.00, *p* < .001. The size of the effect was small *r* = .200. See Fig. 1, top-right panel.

#### CRT-PI scores

The CRT-PI scores did not differ significantly for endorsers (*M* = 0.73, *Mdn* = 0.80, *SD* = 0.33) and non-endorsers (*M* = 0.70, *Mdn* = 0.83, *SD* = 0.38), *W* = 40256.00, *p* = .839, *r* = .008. See Fig. 1, bottom-left panel.

#### CRT response time

**Fig. 2:**
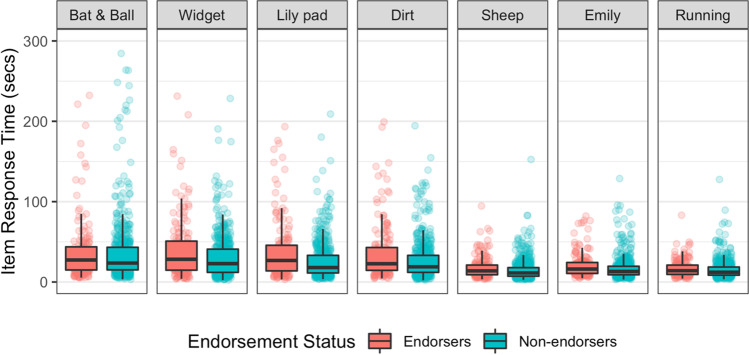
Box and whisker plots showing the Cognitive Reflection Test (CRT) Item Response Time for each CRT item by implausible belief endorsement status. The box depicts the median, 25th and 75th percentiles. The whiskers extend from the hinge to the value no further than 1.5 x interquartile range above and below the upper and lower hinges. Outliers are represented by black dots. *Note:* For clarity of data representation, extreme responses (> 300 s) are not shown in this figure. Group differences were found for all items except the ‘Bat and Ball’ problem

The total time taken to complete all CRT items was significantly longer for endorsers (*M* = 212.16 s, *Mdn* = 182.59 s, *SD* = 132.10 s) than for non-endorsers (*M* = 171.61 s, *Mdn* = 143.85 s, *SD* = 108.75 s), with a median difference of 38.7 s between the groups, *W* = 48706.00, *p* = <.001. The size of the effect was small *r* =.147. See Fig. 1, bottom-right panel. The same pattern of significant differences was observed for six of the seven CRT items (see Fig. 2). The difference between the median for endorsers and non-endorsers was significant for all but the Bat and ball item. All effect sizes were small (all *r*s ≤ 0.146).

#### Self-reported decision strategies

Endorsers were significantly less likely to report an intuitive (‘jumped out’) decision strategy than non-endorsers (32.1% vs. 42.2% respectively) *χ*^2^_(1)_ = 5.250, p = .022, but there was no significant difference between the groups in reporting a deliberative (‘puzzled over’) approach (72.8% vs. non-endorsers 72.9%), *χ*^2^_(1)_ = 0.000, *p* = .986. Endorsers were also significantly less likely to report having relied on their memory for some answers (27.2%) than non-endorsers (40.6%) *χ*^2^_(1)_ = 9.507, *p* = .002.

### Post-hoc analyses

#### Analysis 1

The significant association between endorsement status and ‘remembering’ raises the possibility that familiarity with the CRT may be differentially affecting the performance of endorsers and non-endorsers. When comparing CRT-Reflective scores for those who ‘remembered’ (*M* = 4.95, *Mdn = 5, SD = 1.64*) versus those who did not (*M* = 3.59, *Mdn = 4, SD = 1.92*), we found a statistically significant difference, *W* = 44207, *p* < .001, so we reran our primary analyses excluding all participants who reported remembering answers to the CRT. Analysis of this sample (118 endorsers and 298 non-endorsers) produced the same pattern of results for the four primary dependent variables as the preregistered analysis (see Table 2).

#### Analysis 2

To examine whether our results were affected by how ‘endorsers’ and ‘non-endorsers’ were defined, we reran the analyses with more stringent cut-offs; those who rated any one of the *four* implausible claims ≥ 75 were classified as endorsers and non-endorsers were those who rated all four implausible claims ≤ 20. We also removed those who used the internet or remembered their CRT answers, leaving a sample of 129 endorsers and 230 non-endorsers. The results for the four primary dependent variables replicated the results from the preregistered analysis (see Table 2).

#### Analysis 3

To examine the robustness of our results we also applied a lenient definition of endorsement status by classifying participants using only responses to the Prolific pre-screening questions (i.e., comparing those who believe in climate change to those who do not). After removing those who remembered or used the internet to find answers, the sample consisted of 272 endorsers and 223 non-endorsers. The results for the four primary dependent variables replicated the results from the preregistered analysis (see Table 2).

### Exploratory analyses

#### Non-intuitive incorrect scores

Whereas the Reflective and Intuitive scores tally the number of correct or lure responses participants provide, respectively, it is also possible for participants to give non-intuitive incorrect answers. We explored whether endorsers and non-endorsers differed on this metric. Indeed, endorsers gave significantly more non-intuitive incorrect responses (*M* = 0.70, S*D* = 0.84) than non-endorsers (*M* = 0.51, *SD* = 0.71), *W* = 45539, *p* = .009, *r* = .101.

#### Decision strategies and CRT performance

A comparison of decision strategies across the entire sample showed that those who reported a ‘jumped out’ decision strategy were significantly quicker to respond (W = 85786, *p* = .004), and were more accurate (W = 69662, *p* = .029), than those who did not report using this strategy. Those who reported a ‘puzzled over’ approach took significantly longer to respond (W = 45035, *p* <.001), but there was no significant difference in accuracy between those who did and did not report this strategy (W = 67905, *p* = .174).

#### Relationships between variables

To examine the relationships between variables, we conducted a series of non-parametric correlations between the dependent measures (see Table 3). The Reflective score and Intuitive score were highly negatively correlated. Deliberation time was positively correlated with Intuitive scores but negatively correlated with Reflective scores. The PI score was weakly negatively correlated with the Reflective score, but moderately positively correlated with the Intuitive score, suggesting that it measures intuitive thinking largely independently of Reflective score.

**Table 3:** Spearman Rho correlations between the dependent variables

	Reflective score	Intuitive score	PI score
**Reflective score**	–		
**Intuitive score**	–.920**	–	
**PI score**	–.220**	.510**	–
**Total time**	–.100*	.088*	.049

* < .05, ** < .001

## Discussion

People who believe implausible claims have been described as miserly, reflexive thinkers who follow their intuition rather than engaging in effortful analytical processing (Ballová Mikušková & Čavojová, 2020; Pennycook & Rand, 2019; Shenhav et al., 2012). These characterizations are often based on associations between performance on the CRT and measures of implausible beliefs (e.g., religious thinking, paranormal accounts, fake news and/or conspiracy theories; Patel et al., 2019). Here we tested whether the miserly account adequately explains why people who endorse implausible claims perform worse than non-endorsers on the CRT.

We compared CRT-Reflective, -Intuitive, and -Proportion Intuitive scores and response times of those who strongly believed at least one of three implausible claims to those who disbelieved all three implausible claims. We expected that those who believed the implausible claims would perform worse on the CRT than non-endorsers (lower Reflective scores and higher Intuitive scores). We further reasoned that if endorsers engage effortfully in the task, then they should not make proportionally more incorrect intuitive mistakes than non-endorsers, nor complete the CRT faster than non-endorsers.

In line with prior studies, we found that endorsers of implausible claims had lower Reflective scores than non-endorsers and responded with incorrect intuitive answers more often than non-endorsers. This result is not surprising. A recent meta-analysis has shown a consistent small-to-medium negative association between conspiracy beliefs and various measures of reflective thinking like the CRT (Yelbuz et al., 2022). These findings seemingly suggest that endorsers have a more miserly thinking style than non-endorsers. However, the cognitive effort that the Reflective score supposedly measures is likely confounded by cognitive ability (see Blacksmith et al., 2019; Thomson & Oppenheimer, 2016; Stupple et al., 2017), and the Intuitive score does little more than reverse the sign of the Reflective score. Counter to the miserly hypothesis, we found no difference in the groups’ proportion of incorrect intuitive responses relative to all incorrect responses, which arguably measures intuitive responding in a way that is less impacted by ability. Endorsers were also slower to complete the CRT than non-endorsers. Moreover, although we dichotomized participants into groups, thus sacrificing statistical power, our results were largely consistent irrespective of how we defined endorsement status. These results are inconsistent with the theory that endorsers are more intuitive, reflexive thinkers than non-endorsers.

Our findings for participants’ self-reported decision strategies further challenge the miserly hypothesis. A drawback of the CRT is that it captures the result of cognitive processing and not how a person reached the result (Blacksmith et al., 2019). Indeed, accuracy on many cognitive tasks may not perfectly reflect the underlying mental processes and effort involved, whereas response time more directly measures depth of processing (Alter et al., 2013). A unique insight of our study was that we probed the strategies and sources participants used to complete the CRT. We found that endorsers were not more likely than non-endorsers to report using an intuitive decision strategy, nor less likely to use an analytic decision strategy. The validity of these self-reports was supported by completion time data showing that those who reported an intuitive decision strategy completed the CRT faster than those who did not. Our findings in their totality provide compelling evidence that people who endorse implausible beliefs do not perform worse on the CRT than non-endorsers because they are cognitive misers.

Correlations between implausible belief and CRT performance have been used in the past to infer that implausible beliefs result from lazy thinking (Pennycook & Rand, 2019; 2020; Shenhav et al., 2012; Toplak et al., 2011). However, endorsers of implausible beliefs in our study performed poorly despite deliberating longer on each question. As others have suggested (Blacksmith et al., 2019; Patel et al., 2019; Pennycook et al., 2016; Stanovich, 2018; Stupple et al., 2017), it may be erroneous to assume that poorer overall performance necessarily reflects a miserly or intuitive thinking style. Belief in implausible claims therefore may not be best reduced simply by encouraging more analytical thinking, as is sometimes suggested (e.g., Bago et al., 2020; Bronstein et al., 2019; Lewandowsky, 2021; Pennycook et al., 2020; 2021; Pennycook & Rand, 2019; 2020; Swami et al., 2014). At the same time, we cannot rule out that endorsers of implausible beliefs are lazy when evaluating other claims, such as those relating to topics like vaccines and climate change. Nor can we make strong causal claims given the quasi-experimental nature of the present study.

Looking across our sample, we found that those who deliberated longer tended to have lower Reflective scores and higher Intuitive scores. As others have argued (e.g., Bago & De Neys, 2019), accuracy on the CRT may not always involve overriding an incorrect intuitive answer. Instead, the correct ‘analytic’ answer may come intuitively to some, whereas others may deliberate for a long time and never reach the correct solution. In fact, a ‘jumped out’ decision strategy in this study was associated with higher accuracy. The number of correct responses on the CRT appears to be a poor proxy for how much effort a person has devoted to the task.

A plausible alternative explanation to the miserly account is that the CRT largely captures individual differences in mindware – mental knowledge and abilities – such as general intelligence, working memory, numeric ability, and insight problem-solving ability (Blacksmith et al., 2019; Raoelison et al., 2020; Patel et al., 2019; Stanovich, 2018; Stupple et al., 2017; Teovanovic et al., 2015; Thomson & Oppenheimer, 2016; Toplak et al., 2011). Endorsers and non-endorsers may therefore differ largely in terms of their cognitive faculties rather than their effort. There are two pieces of evidence from the present study that potentially support this mindware explanation. Endorsers in our sample were less educated than non-endorsers, which aligns with other studies (Scherer et al., 2021; van Prooijen, 2017). Endorsers also provided more non-intuitive incorrect answers (i.e., not lure responses) than non-endorsers, suggesting flawed reasoning.

If CRT scores capture a difference between endorsers and non-endorsers in terms of cognitive mindware, then this gap may explain how endorsers come to believe implausible claims in the first place (Rizeq et al., 2021; Scherer et al., 2021; Ståhl & van Prooijen, 2018). People who are more persuaded by fake news, misinformation, the paranormal, conspiracy theories, and/or pseudoscience may not have developed or been taught the skills that support normative evaluations of implausible claims, including mainstream rules and expectations about evidence quality, knowledge, and persuasion (Lewandowsky et al., 2017; Ståhl & van Prooijen, 2018) as well as scientific and critical thinking skills (Dyer & Hall, 2019; Pennycook et al., 2020). Further research is needed to establish clearer links between the CRT, implausible beliefs, and mindware deficits. Several other explanations for why people hold implausible beliefs also exist, including motivated reasoning and distrust of conventional sources (Scherer et al., 2021), but these accounts are not mutually exclusive.

In the present study, we found little evidence that those who endorse implausible claims behave like cognitive misers. People who believe in vaccine conspiracies, a flat earth, or that global warming is a hoax, performed more poorly on the CRT overall than non-endorsers and more frequently responded with the incorrect intuitive answer. However, endorsers took *more* time to respond than non-endorsers and were not more likely to produce proportionally more intuitive mistakes than non-intuitive mistakes. It may therefore be unwise to infer from their comparatively poor performance on the CRT that those who believe implausible claims do so because they fail to expend the requisite cognitive effort. Instead, future research and interventions aimed at minimizing the harms associated with implausible beliefs may need to consider the role and mitigation of motivation, epistemic distrust, and mindware gaps.

### Supplementary Information

Below is the link to the electronic supplementary material.

Supplementary file1 (DOCX 27 KB)

## Data Availability

The datasets generated and analyzed during the current study are available in the Open Science Framework repository at: https://osf.io/uyw98/.

## Abbreviations

CRT: Cognitive Reflection Test
DV: Dependent Variable
MANOVA: Multivariate Analysis of Variance
PI: Proportion Intuitive

## Code availability

The code to analyze the data is available in the Open Science Framework repository at: https://osf.io/uyw98/.
