# Supplementary material for: Thinking false and slow: Implausible beliefs and the Cognitive Reflection Test
Source: Psychon Bull Rev. 2023 Jun 27;30(6):2387–96. doi: 10.3758/s13423-023-02321-2 (PMC10728225; doi:10.3758/s13423-023-02321-2)
Supplement: Supplementary file 1 — Supplementary file1 (DOCX 27 KB) [file 13423_2023_2321_MOESM1_ESM.docx]

**Supplementary materials**

1. **Primary analyses based on liberal endorsement criteria**

Table 1.1. displays the results for comparisons between endorsers and non-endorsers as grouped based on highly liberal criteria. Endorsers were classified as those who rated any of the three beliefs (vaccines, climate change, flat earth) equal to or greater than 10 from 0 (not at all) to 100 (Definitely). Non-endorsers were those participants who rated all items less than 10 out of 100. These results are similar to the analyses reported in the paper.

**Table 1.1 . Analyses based on liberal criteria for endorsement.**

| Endorser Definition: | Any 3 claims ≥ 10 |
| --- | --- |
| Non-Endorser Definition: | All 3 claims < 10 |
| Additional Exclusions: | Internet or remembered |
| Descriptives | Endorser; Non-Endorser |
| n | 272; 223 |
| Gender (% Male) | 50.0; 48.0 |
| Χ^2^ Statistic [p] | χ^2^_(1)_ = 0.085 [p =.770] |
| Highest Education (% Secondary) | 29.4; 22.0 |
| Χ^2^ Statistic [p] | χ^2^_(1)_ = 3.308 [p =.069] |
| English First Language (% Yes) | 66.5; 69.5 |
| Χ^2^ Statistic [p] | χ^2^_(1)_ = 0.493 [p =.483] |
| Age (Mean [SD]) | 39.8 [14.6]; 38.4 [12.7] |
| t Statistic [p] | t_(490.9)_ = 1.159 [p =.247] |
| Hypothesis Testing | |
| CRT-Reflective (Median) | 3; 4 |
| W Statistic [p] r | W = 24020 [p < .001] r =.181 |
| CRT-Intuitive (Median) | 3; 2 |
| W Statistic [p] r | W = 35917 [p <.001] r =.161 |
| CRT-PI (Median) | 0.83; 0.83 |
| W Statistic [p] r | W = 30791 [p =.759] r =.014 |
| CRT-Total Time (Median) | 177.96; 153.02 |
| W Statistic [p] r | W = 3493 [p = .004]  r =.131 |
| Secondary Analysis | |
| ‘Puzzled’ (% Yes) | 76.10; 80.27 |
| Χ^2^ Statistic [p] | χ^2^_(1)_ = 1.239 [p =.266] |
| ‘Jumped’ (% Yes) | 36.8; 37.9 |
| Χ^2^ Statistic [p] | χ^2^_(1)_ = 0.378 [p =.538] |

Shaded cells are significant at p < .05

1. **Response Time analyses after transformations**

To test whether the results for response time depended on parametric of non-parametric analyses, we conducted several parametric tests after transforming the response time data.

- 1. Log transformation: Endorsers (*M* = 5.18) were slower than non-endorsers (*M* = 4.98), *t*(255.5) = 3.618, *p* <.001. The response time data after this transformation were roughly normally distributed.
  2. Square root transformation: Endorsers (*M* = 13.93) were slower than non-endorsers (*M* = 12.57), *t*(255.5) = 3.682, *p* <.001. The result was similar when comparing the groups using a Wilcoxon Rank Sum test, *W* = 48706, *p* < .001.

1. **Bayesian Analyses for each variable**

**Table 3.1. Bayesian t-test for differences between endorsers and non-endorsers (as defined in preregistration).**

| Outcome variables | BF |
| --- | --- |
| Reflective score | >10,000 |
| Intuitive score | >10,000 |
| Non-intuitive incorrect score | 6.25 |
| PI score | 0.15 |
| Response time | 155.57 |
| Log(Response time) | 93.99 |

1. **Linear models**

We conducted several linear models to establish which variables predicted implausible beliefs with all variables thrown in. For each model, we used the preregistered criteria for endorsement. Only Reflective scores and response time were consistent predictors across each model.

**Table 4.1. Linear model with the mean of the three implausible items as the DV and all predictors.**

|  | IB mean | | |
| --- | --- | --- | --- |
| Predictors | Estimates | CI | p |
| (Intercept) | 22.17 | 8.64 – 35.70 | **0.001*** |
| reflective score | -2.18 | -4.34 – -0.01 | **0.049*** |
| intuitive score | 0.93 | -1.75 – 3.61 | 0.495 |
| pi score | -3.44 | -8.99 – 2.12 | 0.225 |
| total time | 0.03 | 0.02 – 0.04 | **<0.001*** |
| strat puzzled [1] | -2.41 | -5.73 – 0.91 | 0.155 |
| strat jumped [1] | -1.99 | -5.00 – 1.02 | 0.194 |
| Observations | 800 | | |
| R^2^ / R^2^ adjusted | 0.095 / 0.088 | | |

**Table 4.2. Linear model with the mean score for the four implausible items as the DV and all predictors.**

|  | IB mean | | |
| --- | --- | --- | --- |
| Predictors | Estimates | CI | p |
| (Intercept) | 24.56 | 11.65 – 37.47 | **<0.001*** |
| reflective score | -2.61 | -4.68 – -0.55 | **0.013*** |
| intuitive score | 0.61 | -1.95 – 3.17 | 0.640 |
| pi score | -3.55 | -8.85 – 1.75 | 0.189 |
| total time | 0.03 | 0.02 – 0.04 | **<0.001*** |
| strat puzzled [1] | -2.71 | -5.88 – 0.46 | 0.094 |
| strat jumped [1] | -1.96 | -4.82 – 0.91 | 0.181 |
| Observations | 800 | | |
| R^2^ / R^2^ adjusted | 0.110 / 0.103 | | |

**Table 4.3. Generalized additive model with the mean for the three implausible items as the DV and all predictors. Tweedie was specified as distribution family.**

|  | IB mean | | |
| --- | --- | --- | --- |
| Predictors | Estimates | CI | p |
| (Intercept) | 23.14 | 9.14 – 58.58 | **<0.001*** |
| reflective score | 0.87 | 0.74 – 1.01 | 0.063 |
| intuitive score | 1.05 | 0.87 – 1.27 | 0.616 |
| pi score | 0.83 | 0.54 – 1.28 | 0.401 |
| total time | 1.00 | 1.00 – 1.00 | **<0.001*** |
| strat puzzled [1] | 0.85 | 0.67 – 1.08 | 0.191 |
| strat jumped [1] | 0.89 | 0.71 – 1.10 | 0.278 |
| Observations | 800 | | |
| R^2^ | 0.080 | | |

**Table 4.4. Generalized additive model with the mean for the four implausible items as the DV and all predictors. Tweedie was specified as distribution family.**

|  | IB mean | | |
| --- | --- | --- | --- |
| Predictors | Estimates | CI | p |
| (Intercept) | 25.27 | 10.39 – 61.42 | **<0.001*** |
| reflective score | 0.84 | 0.73 – 0.98 | **0.023*** |
| intuitive score | 1.04 | 0.87 – 1.24 | 0.700 |
| pi score | 0.83 | 0.54 – 1.26 | 0.372 |
| total time | 1.00 | 1.00 – 1.00 | **<0.001*** |
| strat puzzled [1] | 0.84 | 0.67 – 1.06 | 0.150 |
| strat jumped [1] | 0.89 | 0.72 – 1.10 | 0.278 |
| Observations | 800 | | |
| R^2^ | 0.097 | | |
